# Association between sexual dysfunction and symptom burden in women with Chronic Obstructive Pulmonary Disease

**DOI:** 10.1371/journal.pone.0357921

**Published:** 2026-09-15

**Authors:** Müfide Arzu Özkarafakılı, Cemil Kutsal, Nihat Türkmen, Hatice Yılmaz

**Affiliations:** 1 Department of Chest Diseases, Şişli Hamidiye Etfal Training and Research Hospital, University of Health Sciences, Istanbul, Turkey; 2 Department of Urology, Şişli Hamidiye Etfal Training and Research Hospital, University of Health Sciences, Istanbul, Turkey; 3 Department of Obstetrics and Gynecology, Şişli Hamidiye Etfal Training and Research Hospital, University of Health Sciences, Istanbul, Turkey; Tarbiat Modares University Faculty of Medical Sciences, IRAN, ISLAMIC REPUBLIC OF

## Abstract

**Objectives:**

To investigate sexual dysfunction as a clinically relevant but under-recognized manifestation of disease burden in women with COPD and to identify its clinical and psychological determinants.

**Study design:**

In this cross-sectional study, 100 consecutive women with stable COPD (mean age 57 ± 7 years) attending an outpatient chest clinic were enrolled.

**Main outcome measures:**

Participants underwent pulmonary function testing and completed the COPD Assessment Test (CAT), Modified Medical Research Council (mMRC) scale, Beck Depression Inventory, Short Form-36 (SF-36), Functional Assessment of Chronic Illness Therapy–Fatigue (FACIT-FS), and Female Sexual Function Index (FSFI).

**Results:**

Sexual dysfunction (FSFI ≤26.55) was more prevalent in patients with greater disease burden, particularly those in GOLD stage E, and was associated with significantly higher fatigue and depressive symptoms. FSFI scores showed a positive correlation with FACIT-FS and quality-of-life domains, and a negative correlation with depression scores (all p < 0.05). In multivariable analysis, fatigue was positively associated with FSFI scores, whereas depression and body mass index were negatively associated. Patients with more severe disease (GOLD E), comorbidities, and dyspnea during intercourse had lower FSFI scores, while those receiving triple therapy demonstrated higher scores.

**Conclusions:**

Sexual dysfunction was associated with greater symptom burden in women with COPD and was closely related to fatigue and depression. Assessment of sexual function may contribute to a more comprehensive evaluation of disease burden in clinical practice.

## Introduction

Chronic Obstructive Pulmonary Disease (COPD) is a progressive and treatable condition characterized by persistent respiratory symptoms, including dyspnea, cough, and sputum expectoration [1]. It ranks as the seventh leading cause of disabilities worldwide, and the ongoing respiratory symptoms diminish the ability to perform daily activities and negatively impact an individual’s emotional well-being [1,2].

COPD is known to affect more men than women; nevertheless, global studies suggest that in some European countries, it is increasing in women, despite a decreasing trend in men [3]. While chronic respiratory symptoms and exacerbations primarily degrade the quality of life in patients with COPD, most of these individuals are in late adulthood and deal with several comorbidities [4]. Despite being overlooked, it has a significant impact on sexual health since it results in depressive symptoms [5]. Furthermore, patients with COPD frequently experience growing fatigue and lack of energy that influence their exercise tolerance and psychological status and compromise the family routines accompanied by limitations in sexual function [6].

Fatigue has been shown to be closely associated with depression and anxiety in women with COPD [7]. In addition, the progressive nature of COPD may increase fear of breathlessness, reduce sexual desire, and lower self-esteem over time [8]. However, sexual dysfunction in COPD has traditionally been regarded primarily as a male issue, leaving the sexual health concerns of women under-recognized and under-investigated [9].

Female sexual dysfunction is defined as a persistent or recurrent disturbance in sexual desire, arousal, orgasm, or pain associated with significant personal distress [10]. Although sexual dysfunction appears to be more prevalent in women than in men, studies specifically evaluating women with COPD remain limited [9,11]. Most previous studies have primarily reported the prevalence of sexual dysfunction or included mixed-sex populations, providing limited information on women with COPD. Consequently, the relationships between sexual dysfunction and important components of COPD-related symptom burden, including fatigue, depression, and disease severity, remain insufficiently characterized.

Given that many patients remain symptomatic despite optimal bronchodilator therapy, a more comprehensive evaluation of disease burden is warranted [1]. In this context, sexual dysfunction may represent an additional aspect of COPD-related symptom burden that is not captured by conventional respiratory assessments.

Therefore, the aim of this study was to investigate sexual dysfunction as a component of overall disease burden in women with COPD and to examine its association with clinical severity, fatigue, and depression.

## Materials and methods

### Study design and population

This cross-sectional study was conducted in the outpatient chest clinic of a university hospital to evaluate sexual dysfunction as a component of disease burden in women with COPD. The study is reported in accordance with the Strengthening the Reporting of Observational Studies in Epidemiology (STROBE) Statement.

Female patients aged ≥18 years with a confirmed diagnosis of COPD according to the Global Initiative for Chronic Obstructive Lung Disease (GOLD) criteria were consecutively recruited between 30/10/2023 and 25/12/2025 [1]. All patients had been followed in the outpatient clinic for at least three years and were clinically stable at the time of inclusion, defined as the absence of exacerbations, systemic corticosteroid use, or respiratory tract infections within the preceding two months.

Only sexually active women who provided informed consent were included. Sexual activity was confirmed through a structured physician-administered interview, and patients reporting at least one sexual encounter within the previous four weeks were considered eligible. Exclusion criteria included pregnancy or postpartum status within the previous six months, prior gynecological surgery, uncontrolled cardiovascular disease or uncontrolled diabetes mellitus, malignancy, endocrine disorders, and, neurological, or cognitive disorders. Uncontrolled cardiovascular disease or diabetes mellitus was defined as physician-documented unstable disease requiring hospitalization, emergency department admission or treatment escalation at the time of study enrollment.

Patients with a documented current or past psychiatric disorder requiring psychiatric treatment, as determined from the medical records were excluded. All patients underwent a gynecological evaluation prior to study procedures.

We also inquired about the patients’ medical history regarding pre-existing comorbidities such as allergic rhinitis and asthma for exclusion purposes. Participants were either nonsmokers or former smokers who had quit smoking for at least one year. The individuals were stable and had no history of exacerbations, systemic corticosteroid administration, or respiratory tract infections in the preceding two months. Clinical data including inhaled bronchodilator therapy, oxygen treatment, and ventilatory support were recorded. Treatment adherence and inhaler technique were assessed by the physician at the time of evaluation.

### Study measures

Following a detailed physical examination, sociodemographic information, clinical data, and treatment details were all recorded for all participants. Pulmonary function tests were performed by an experienced technician in accordance with the American Thoracic Society guidelines [12]. Spirometric measurements were obtained using the MIR Spirolab II device (Rome, Italy), including forced expiratory volume in one second (FEV1), forced vital capacity (FVC), and the FEV1/FVC ratio. COPD was defined by a post-bronchodilator FEV1/FVC ratio <70% [1]. Disease severity was classified according to GOLD criteria based on predicted FEV1 values as mild (≥80%, GOLD 1), moderate (50–79%, GOLD 2), severe (30–49%, GOLD 3), and very severe (<30%, GOLD 4) [1].

Symptom burden was assessed using the COPD Assessment Test (CAT), a validated instrument ranging from 0 to 40, with higher scores indicating greater symptom severity [1]. The validated Turkish version of the CAT was used in this study [13]. Dyspnea severity was evaluated using the Modified Medical Research Council (mMRC) scale, which grades dyspnea from 0 to 4, with higher scores indicating greater functional limitation [1].

Clinical data regarding exacerbation history and treatment in the previous year were obtained from electronic medical records. Patients were categorized into GOLD A, B, and E groups by integrating symptom severity and exacerbation risk according to current GOLD recommendations [1]. Depressive symptoms were assessed using the Beck Depression Inventory (BDI), a 21-item self-administered questionnaire evaluating depressive symptoms over the preceding week [14,15]. Patients are asked to respond on a four-point scale, in which the total score is between 0–63; with 10–16 indicating mild depression, 17–29 indicating moderate depression, and 30–63 indicating severe depression.

Health-related quality of life was assessed using the Short Form-36 (SF-36) questionnaire, a validated instrument comprising 36 items across eight domains [16]. The SF-36 generates two summary measures: the Physical Component Summary and the Mental Component Summary. Higher scores indicate better health status, whereas lower scores reflect greater impairment. The validated Turkish version of the SF-36 was used in this study [17].

Fatigue was evaluated using the Functional Assessment of Chronic Illness Therapy–Fatigue scale (FACIT-FS), a 13-item self-reported measure assessing fatigue severity and its impact on daily activities and emotional well-being [18].

Each item is scored on a 5-point Likert scale (0–4), yielding a total score ranging from 0 to 52, with higher scores indicating lower levels of fatigue. The FACIT scale is found to be reliable and valid for determining COPD fatigue [18].

Sexual function was assessed using the Female Sexual Function Index (FSFI), a validated 19-item questionnaire evaluating sexual function over the preceding four weeks across six domains: desire, arousal, lubrication, orgasm, satisfaction, and pain [19]. The scores range from 0 to 5 for each domain weighting, and the total score for sexual function has a maximum of 36. While higher scores correspond to better sexual functioning, a cutoff score of 26.55 on the FSFI is determined to indicate sexual dysfunction [10]. We applied the validated Turkish version of the FSFI form for our study's subjects [20]. The survey was completed under the supervision of physicians from the Department of Urology and the Department of Gynecology.

### Ethical considerations

The study protocol was approved by the Institutional Ethics Committee on 10/10/2023 (Approval No. 4125) before data collection. Written informed consent was obtained from all participants before enrollment. Participants were informed about the purpose and procedures of the study, and the confidentiality of their personal information was assured. The study was conducted in accordance with the principles of the Declaration of Helsinki.

### Statistical analysis

Statistical analyses were performed using IBM SPSS Statistics for macOS, version 29.0 (IBM Corp., Armonk, NY, USA). An a priori sample size calculation was performed using G*Power version 3.1. Assuming a two-sided α level of 0.05 and 80% statistical power, the required sample size was estimated as 94 participants for comparisons of continuous variables, 69 participants for chi-square analyses, and 80 participants for multivariable linear regression analysis (effect size *f*² = 0.20). Since 100 women with COPD were included, the final sample exceeded the minimum required sample size for all planned analyses.

Categorical variables were expressed as frequencies and percentages, while continuous variables were presented as mean ± standard deviation or median (minimum–maximum), as appropriate. The normality of data distribution was assessed using the Kolmogorov–Smirnov test.

For comparisons between two groups, the independent samples t-test was used for normally distributed variables, and the Mann–Whitney U test was applied for non-normally distributed variables. Categorical variables were compared using the Chi-square test or Fisher’s exact test, as appropriate. Correlation analyses between FSFI scores and clinical variables were performed using Pearson or Spearman correlation coefficients, depending on data distribution.

To identify factors independently associated with FSFI scores, multivariable linear regression analysis was performed. Candidate variables were selected based on clinical relevance and/or statistically significant associations in univariable analyses and included age, body mass index, GOLD stage, FEV1, FVC, FEV1/FVC ratio, CAT score, mMRC score, FACIT-FS score, Beck Depression Inventory score, COPD duration, comorbidity, treatment category, dyspnea during intercourse, fatigue during intercourse, and SF-36 domains. Given the relatively large number of clinically plausible predictors in relation to the sample size, a backward stepwise procedure was used as an exploratory variable-selection method to derive a parsimonious model. Regression results were reported as unstandardized coefficients (B), standardized coefficients (β), 95% confidence intervals (95% CI), and corresponding p-values. Model performance was assessed using R², adjusted R², and the overall F statistic. Multicollinearity was assessed using variance inflation factors (VIF) and tolerance values. A two-sided *p*-value <0.05 was considered statistically significant.

## Results

### Characteristics of the study population

A total of 100 women with COPD were included in the study, with a mean age of 57 ± 7 years (range: 40–65). Patients with incomplete data or those who did not meet the study requirements were excluded.

Comorbidities were present in 66% of the participants. The most common comorbid conditions were hypertension (40.9%), diabetes mellitus, and coronary artery disease (34.8%).

Regarding sociodemographic characteristics, 43% of the participants had a university degree, 63% were employed, and 66% were living in urban areas.

Most patients (86%) reported having a regular partner. Dyspnea during intercourse was reported by 49% of participants, while 47% reported experiencing fatigue.

### Association between FSFI scores and the clinical variables

Consistent with previous studies, an FSFI score ≤26.55 was used to define female sexual dysfunction [20].

Comparisons of demographic and clinical characteristics according to FSFI categories are presented in Table 1,2. Sexual dysfunction was more frequently observed in patients with greater disease burden, particularly those classified as GOLD stage E, whereas patients without dysfunction were more commonly in GOLD stage B (p = 0.018).

**Table 1:** Comparison of demographic and clinical features of patients according to FSFI scores.

Variables (n=100)	FSFI>26.55 (n=19) n (%) or Median (Min-Max)	FSFI≤26.55 (n=81) n (%) or Median (Min-Max)	p-value
**Age/years**	57 (40-65)	58 (43-65)	0,293
**Body mass index kg/m^2^**	27.1 (21-42.1)	26.7 (20.1-41.8)	0.629
**Comorbidity**	12 (63.2)	54 (66.7)	0.983
Coronary artery diseases	4 (33.3)	19 (35.2)	1.000
Hypertension	6 (50)	21 (38.9)	0.528
Diabetes Mellitus	6 (50)	17 (31.5)	0.316
Chronic renal diseases	1 (8.3)	2 (3.7)	0.458
**β-2 mimetic use before intercourse**	11 (57.9)	48 (59.3)	1.000
**Partner status**			0.460
Single	4 (21.1)	10 (12.3)	
Living with a spouse	15 (78.9)	71 (87.7)	
**Employment**			1.000
Unemployed	7 (36.8)	30 (37)	
Employed	12 (63.2)	51 (63)	
**Education**			0.230
Non-university graduates	8 (42.1)	49 (60.5)	
University graduates	11 (57.9)	32 (39.5)	
**Residence area**			0.983
Suburban	7 (36.8)	27 (33.3)	
Urban	12 (63.2)	54 (66.7)	
Dyspnea during intercourse	4 (21.1)	45 (55.6)	**0.014**
Fatigue during intercourse	9 (47.4)	38 (46.9)	1.000
**FACIT-FS score**	42 (30-49)	38 (22-46)	**0.031**
**BECK Depression Inventory**	16 (7-27)	18 (10-31)	0.187
**SF-36 scale**			
Physical Component Summary	**70.6 (36.88-87.50)**	**70 (30.63-88.75)**	**0.549**
Mental Component Summary	73.25 (39.45-86.50)	71.38 (29.88-89.50)	0.788

FSFI: Female Sexual Function Index, FACIT-FS: The Functional Assessment of Chronic Illness Therapy Fatigue Scale, SF-36: Short Form 36. Effect size was expressed as Cohen’s d (0.59) for FACIT-FS.

**Table 2:** Comparison of clinical features of patients according to FSFI scores.

Variables (n=100)	FSFI>26.55 (n=19) n (%) or Median (Min-Max)	FSFI≤26.55 (n=81) n (%) or Median (Min-Max)	p-value
**GOLD Stages**			**0.018**
A	1 (5.3)	14 (17.3)	
B	13 (68.4)	27 (33.3)	
E	5 (26.3)	40 (49.4)	
**FEV**_**1**_ **% Stages**			0.742
Stage-I	0 (0)	3 (3.7)	
Stage-II	13 (68.4)	51 (63)	
Stage-III	6 (31.6)	25 (30.9)	
Stage-IV	0 (0)	2 (2.5)	
**CAT score**			
CAT < 10	1 (5.3)	4 (4.9)	0.953
CAT ≥ 10	18 (94.7)	77 (95.1)	
**FEV**_**1**_ **%**	59 (39-75)	59 (28-88)	0.738
**FVC%**	80 (55-98)	80 (57-110)	0.340
**FEV**_**1**_ **/FVC%**	70 (59-70)	67 (42-69)	0.481
**mMRC score**	2 (0-3)	2 (0-3)	0.232
**Duration of COPD (years)**	10 (2-28)	10 (2-28)	0.615
**Exacerbations**	13 (68.4)	57 (70.4)	1.000
**Exacerbations (previous year)**	1 (0-3)	1 (0-3)	0.581
**Treatment**			0.353
**LABA+LAMA**	5 (26.3)	27 (33.3)	
**Triple**	13 (68.4)	42 (51.9)	
**LAMA**	1 (5.3)	12 (14.8)	
**Long-term oxygen treatment (LTOT)**	4 (21.1)	16 (19.8)	1.000
**Non-invasive Positive Pressure Ventilation (NPPV)**	5 (26.3)	18 (22.2)	0.764

FSFI: Female Sexual Function Index, FEV1%: predicted value of forced expiratory volume in 1st second, FVC%: predicted value of forced vital capacity, CAT: Chronic Obstructive Pulmonary Disease Assessment Test, mMRC: Modified Medical Research Council, COPD: Chronic Obstructive Pulmonary Disease, LABA: Long-acting beta-2 agonist, LAMA: Long-acting muscarinic antagonist. Effect size was expressed as Cramér's V for GOLD Stage (0.28).

Patients with sexual dysfunction had significantly lower FACIT-FS scores, indicating higher levels of fatigue (p = 0.031). In addition, dyspnea during intercourse was more frequently reported in this group (p = 0.014).

**Fig 1:**
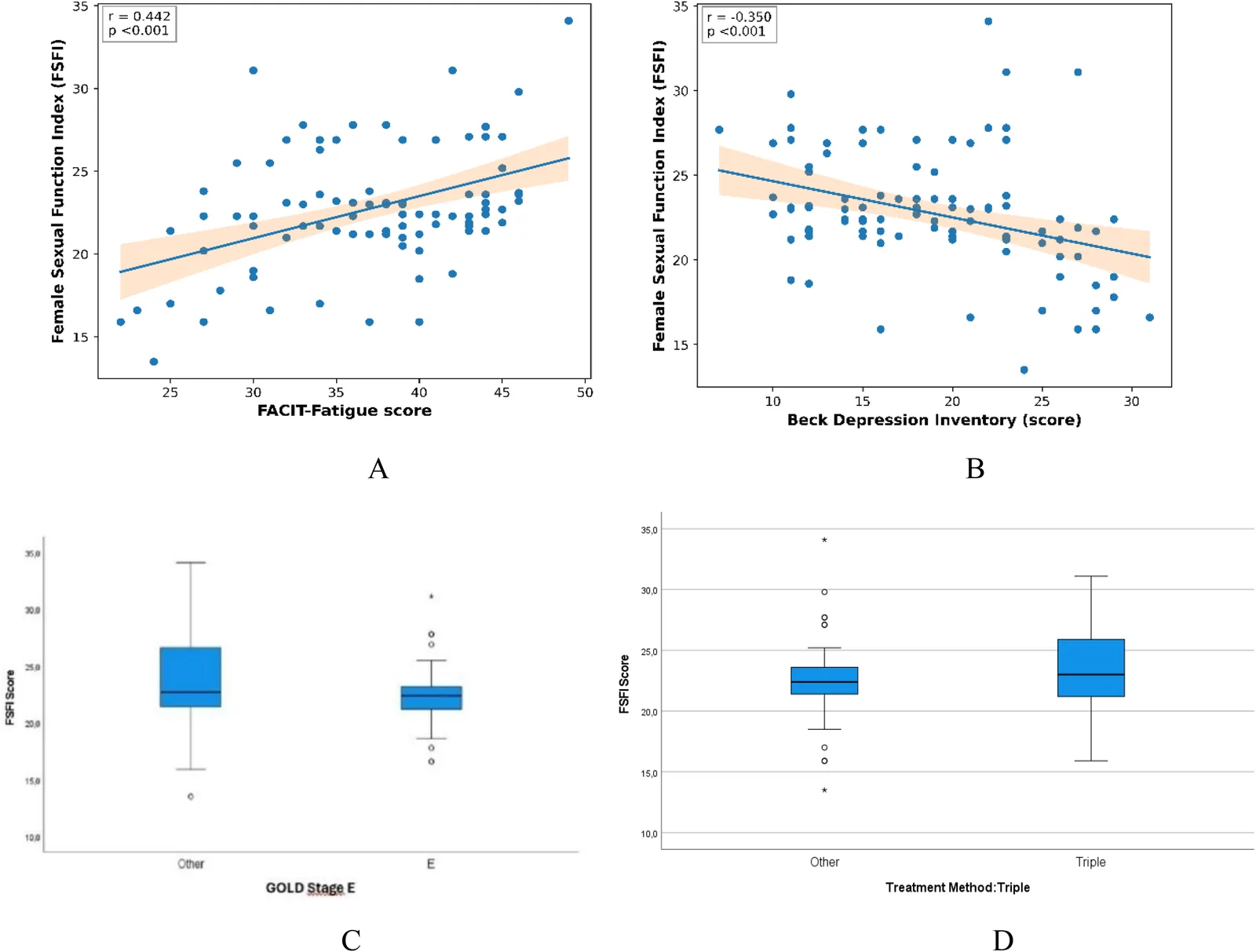
Associations between Female Sexual Function Index (FSFI) scores and clinical characteristics in women with COPD. (A) Scatterplot showing the relationship between FSFI scores and FACIT-FS scores. The solid line represents the fitted linear regression line, and the shaded area indicates the 95% confidence interval. (B) Scatterplot showing the relationship between FSFI scores and Beck Depression Inventory scores. The solid line represents the fitted linear regression line, and the shaded area indicates the 95%confidence interval. (C) Boxplot comparing FSFI scores according to GOLD stage E. (D) Boxplot comparing FSFI scores according to treatment with triple therapy. FSFI: Female Sexual Function Index; FACIT-FS: Functional Assessment of Chronic Illness Therapy Fatigue Scale.

Further correlation analyses between FSFI scores and clinical variables are presented in Table 3. FSFI scores showed a moderate positive correlation with FACIT-FS scores (r = 0.442, p < 0.001), indicating an association between better sexual function and lower levels of fatigue. (Fig 1A). In contrast, FSFI scores were negatively correlated with Beck Depression Inventory scores (r = −0.350, p < 0.001), suggesting that higher depressive symptom burden is associated with poorer sexual function (Fig 1B).

**Table 3:** Correlation analysis of FSFI scores and the variables.

Variable		FSFI Score					
		
		Mean	SD	r	p	%95 CI	
**Age/years**		56.68	6.58	−0.067	0.506	−0.266	0.137
**BMI kg/m** ^**2**^		27.80	4.99	−0.167	0.098	−0.386	0.055
**FEV**_**1**_ **%**		56.63	12.76	0.011	0.911	−0.167	0.181
**CAT**		23.57	8.06	−0.104	0.304	−0.277	0.083
**FACIT-FS**		37.28	6.19	**0.442**	**< 0.001**	**0.232**	**0.607**
**mMRC**		1.94	0.99	−0.047	0.644	−0.228	0.151
**BECK Depression Inventory**		18.62	5.82	**−0.350**	**< 0.001**	**−0.538**	**−0.145**
**Duration of COPD/years**		11.17	6.06	0.081	0.424	−0.084	0.244
**SF-36**							
Physical functioning		59.50	24.13	0.176	0.080	−0.021	0.353
Physical role difficulties		80.25	32.43	**0.229**	**0.022**	**0.049**	**0.397**
Emotional role difficulties		72.32	39.09	**0.226**	**0.024**	**0.040**	**0.378**
Energy-Vitality		51.30	14.52	0.154	0.127	−0.054	0.344
Mental health		62.92	9.61	**0.228**	**0.023**	**0.036**	**0.398**
Social functioning		70.42	20.85	0.159	0.114	−0.028	0.321
Bodily pain		73.80	18.27	0.098	0.334	−0.108	0.278
General health		45.40	14.99	0.096	0.343	−0.086	0.257
**Comorbidity**	–	23.33	3.51	0.003	0.974	−0.094	0.280
	+	22.55	3.58				
**Treatment**	LABA+LAMA	22.34	4.01	0.097	0.336	−0.112	0.285
	Triple	23.04	3.56				
	LAMA	22.95	2.23				

FSFI: Female Sexual Function Index, CI: Confidence Interval, BMI: Body mass index, FEV1%: predicted value of forced expiratory volume in 1st second, CAT: Chronic Obstructive Pulmonary Disease Assessment Test, FACIT-FS: The Functional Assessment of Chronic Illness Therapy Fatigue Scale, mMRC: Modified Medical Research Council, COPD: Chronic Obstructive Pulmonary Disease, SF-36: Short Form-36, LABA: Long-acting beta-2 agonist, LAMA: Long-acting muscarinic antagonist.

Additionally, FSFI scores demonstrated weak but statistically significant positive correlations with SF-36 subdomains, including physical role functioning (r = 0.229, p = 0.022), emotional role functioning (r = 0.226, p = 0.024), and mental health (r = 0.228, p = 0.023) subdomains.

Multivariable linear regression analysis identified factors independently associated with FSFI scores; the results are summarized in Table 4. Before model construction, multicollinearity was assessed. In the final backward stepwise multivariable model, variance inflation factors ranged from 1.08 to 1.47, with corresponding tolerance values ranging from 0.68 to 0.93, indicating that multicollinearity was not a concern (Supplementary S1 Table).

**Table 4:** Multivariable linear regression analysis identifying independent predictors of FSFI scores.

Coefficients^a^							
Model^b^	Standardized coefficients		Unstandardized coefficients	t-value	p-value	%95 Confidence Interval	
	β	St. Error	Beta			Lower limit	Upper limit
Constant	94.445	22.241		4.246	< 0.001	50.239	138.652
BMI kg/m^2^	−0.144	0.054	−0.202	−2.663	0.009	−0.252	−0.037
GOLD Stage E	−3.779	1.010	−0.531	−3.740	< 0.001	−5.787	−1.770
FACIT-FS	0.255	0.053	0.444	4.796	< 0.001	0.149	0.361
BECK Depression Inventory	−0.144	0.051	−0.236	−2.812	0.006	−0.246	−0.042
Dyspnea during intercourse	−1.347	0.585	−0.190	−2.301	0.024	−2.510	−0.184
Comorbidity	−1.270	0.606	−0.169	−2.095	0.039	−2.474	−0.065
Treatment with Triple Therapy	3.604	0.995	0.507	3.622	< 0.001	1.626	5.582

a. Dependent variable: FSFI; R^2^: 0.575; Model: p < 0.001.

b. Backward Model.

FSFI: Female Sexual Function Index, BMI: Body mass index, FACIT-FS: The Functional Assessment of Chronic Illness Therapy Fatigue Scale.

All clinically relevant variables were entered into the multivariable linear regression model, and a backward stepwise selection procedure was used to identify independent predictors of FSFI scores. In the final model, higher FACIT-FS scores were independently associated with higher FSFI scores (p < 0.001) (Fig 1A), indicating better sexual function with lower levels of fatigue. In contrast, higher Beck Depression Inventory scores and body mass index were independently associated with lower FSFI scores (p = 0.006 and p = 0.009, respectively) (Fig 1B).

Patients classified as GOLD stage E had significantly lower FSFI scores compared with other stages (β = −3.779) (Fig 1C). Similarly, the presence of comorbidities (β = −1.270, p = 0.039) and dyspnea during intercourse (β = −1.347, p = 0.024) were independently associated with reduced FSFI scores (p < 0.001).

In contrast, patients receiving triple therapy demonstrated significantly higher FSFI scores compared with other treatment groups (β = 3.604, p < 0.001) (Fig 1D).

## Discussion

This study demonstrates that sexual dysfunction represents a clinically relevant yet under-recognized component of disease burden in women with COPD. A substantial proportion of patients reported sexual complaints, supporting previous findings while further highlighting the multidimensional impact of COPD beyond traditional respiratory outcomes [9].

Our results suggest that sexual dysfunction in COPD is not an isolated phenomenon but is closely linked to key components of disease burden, particularly fatigue and depressive symptoms. In line with prior studies, we observed a significant association between sexual dysfunction and fatigue, as assessed by the FACIT-FS scale [6,21–23]. Notably, nearly half of the participants reported fatigue and dyspnea during sexual activity, indicating that both physical limitation and symptom burden may be associated sexual functioning.

Fatigue, a prevalent yet often under-recognized symptom in COPD, has been consistently associated with reduced functional capacity, increased exacerbation risk, and psychological distress [24,25]. Consistent with these observations, our previous case-control study also identified fatigue during intercourse as the strongest independent correlate of female sexual dysfunction in women with COPD [26]. The present study extends these findings by showing an independent association between fatigue and sexual health outcomes after adjustment for a broader range of clinical and psychological variables, suggesting that COPD-related morbidity encompasses both physiological and psychosocial dimensions.

Dyspnea is the predominant symptom in COPD, significantly limiting physical activity and impairing quality of life [1]. However, the relationship between sexual function and conventional markers of disease severity, such as airflow limitation and spirometric indices, remains inconsistent in the literature [9,23].

In our study, FSFI scores were not significantly associated with spirometric parameters (FEV1), CAT scores, mMRC grades, or GOLD stage classification based solely on airflow limitation. These findings suggest that sexual dysfunction in COPD may not be directly explained by the degree of airflow obstruction alone.

One possible explanation is that commonly used clinical tools, such as CAT and mMRC, primarily capture symptom burden during routine daily activities but may not adequately reflect the physiological and perceptual demands of exertional states, including sexual activity. Sexual function represents a complex interaction of physical capacity, ventilatory limitation, and psychological factors, which may not be fully captured by standard respiratory assessments [5,9].

Consistent with this perspective, we also found no significant association between sexual dysfunction and age, disease duration, or educational status. Taken together, these findings highlight that sexual dysfunction in COPD is a multifactorial phenomenon that extends beyond traditional measures of disease severity. This underscores the need for a more comprehensive clinical approach that incorporates both physiological and psychosocial dimensions of disease burden in routine assessment [27].

Patients with sexual dysfunction were more frequently classified in GOLD stage E, representing a subgroup characterized by higher symptom burden, increased exacerbation risk, and greater healthcare utilization. These findings suggest that sexual dysfunction may be associated with greater overall disease burden.

Exacerbations and frequent healthcare encounters may be associated with poorer sexual function, as previously reported [21]. Since the GOLD 2011 update, COPD assessment has incorporated symptom burden, exacerbation history, and comorbidities to guide treatment decisions [1]. Within this framework, our findings support the notion that sexual dysfunction aligns more closely with overall disease burden rather than airflow limitation alone.

Interestingly, although sexual dysfunction was more common among patients in GOLD stage E, those receiving triple therapy demonstrated higher FSFI scores. This finding may reflect better symptom control among patients receiving optimized pharmacological treatment; however, no causal relationship can be inferred from the present cross-sectional study [28].

In evaluating the appropriate treatment goals for COPD, alleviating dyspnea and preventing exacerbations are the main focus; however, given the considerations of the overlapping contribution of comorbidities and individual factors [1,4].

Multimorbidity is a prevalent health issue in the aging population. Experiencing COPD is far beyond respiratory symptoms only, such as dyspnea or coughing. In the reality of living, those people have to cope with fatigue, anxiety, or depression on a daily basis [24]. Breathlessness, fatigue and depressive symptoms are so intertwined that deciding which one is a cause or a somatic symptom stands as a challenge in follow-up visits. A systematic meta-analysis on sexual health in COPD indicated that COPD had a more significant impact on sexual life than other chronic diseases, such as heart failure or diabetes [8]. Our findings highlighted that depression might be associated with sexual issues in these females, who experienced compromised sexual functioning, aligning with the limited literature on the subject [8].

Female sexual dysfunction is a multifactorial condition in which biological, psychological, and sociocultural factors interact through complex mechanisms [5,29]. In this context, fatigue and depression appear to play a central role in the development and maintenance of impaired sexual function, particularly in domains such as sexual desire and arousal [30,31].

A bidirectional relationship likely exists between these factors. Reduced sexual activity may increase psychological distress, while depressive symptoms and fatigue can further impair sexual motivation and performance. Depression may contribute to diminished energy levels, reduced self-esteem, and impaired body perception, ultimately leading to avoidance of intimate relationships and social withdrawal in women with COPD [30].

In our study, these interrelated mechanisms may explain the observed associations between sexual dysfunction and the physical and emotional role limitations, as well as mental health domains of the SF-36. Importantly, fatigue and depression are potentially modifiable factors in current clinical practice and have been linked to worse health status and increased exacerbation risk in COPD [24,25]. Therefore, their recognition may be essential for a more comprehensive management of disease burden.

Although the overall prevalence of comorbidities was similar between patients with and without sexual dysfunction, the presence of comorbid conditions was associated with lower FSFI scores, consistent with previous reports [23,32].

Hypertension and diabetes mellitus were the most common comorbidities in our cohort. Both conditions are well-established contributors to female sexual dysfunction through shared pathophysiological mechanisms, including endothelial dysfunction, chronic low-grade inflammation, and hormonal imbalance [11,33–35]. In addition, these comorbidities share common risk factors with COPD, which may further amplify their combined impact on overall disease burden [33,35]. Collectively, these mechanisms support the concept that sexual dysfunction in COPD is likely driven by a complex interaction between vascular, inflammatory, physical, and psychological pathways rather than by airflow limitation alone [26].

Similar to male sexual dysfunction, female sexual function is partly dependent on vascular integrity. Endothelial dysfunction and impaired genital blood flow represent key shared mechanisms linking cardiovascular risk factors to female sexual dysfunction [27]. In this context, conditions such as hypertension, diabetes mellitus, coronary artery disease, and metabolic syndrome have been consistently associated with impaired sexual function in women [33,34].

In addition, reduced exercise capacity and increased fatigability—common features of both cardiovascular disease and COPD—may further contribute to impaired sexual function by limiting physical performance and tolerance during sexual activity [27,34–36]. Beyond physiological factors, psychological components, particularly depression, also play a critical role by affecting multiple phases of sexual response, including desire, arousal, and satisfaction [27,33,37].

In our study, higher body mass index was independently associated with lower FSFI scores, supporting previous findings [36]. This association may be partially explained by systemic corticosteroid exposure during exacerbations, as well as reduced physical activity. Moreover, increased body weight may negatively affect body image and self-esteem, thereby reducing sexual motivation and engagement.

The prevalence of sexual dysfunction observed in our cohort (81%) is consistent with previous studies conducted in women with COPD, including our earlier case-control study, which reported a prevalence of 82.9% [26]. In contrast, studies conducted in Turkish women from the general population using the same FSFI cutoff have reported substantially lower prevalence rates, ranging from approximately 43% to 53% [38,39]. These findings suggest that the high prevalence observed in the present study may reflect the additional symptom burden associated with COPD, although the contribution of age and comorbidities cannot be completely excluded.

This observation is consistent with recent evidence showing that female sexual disfunction is highly prevalent across chronic diseases, particularly cardiovascular disease and diabetes, supporting the concept that chronic systemic conditions contribute substantionally to impaired sexual health [33,36].

### Limitations and strengths

This study has several limitations. First, its cross-sectional design precludes causal inferences regarding the relationships between sexual dysfunction and clinical variables. Second, the study was conducted at a single center with a relatively small sample size, which may limit the generalizability of the findings. The multivariable model was developed using backward stepwise regression. Although this exploratory approach was used to derive a parsimonious model, it may produce unstable coefficient estimates and optimistic model performance in relatively small datasets. Therefore, these findings should be interpreted cautiously and require confirmation in larger independent cohorts. Furthermore, because the Female Sexual Function Index (FSFI) is validated for sexually active women, only participants reporting at least one sexual encounter during the preceding four weeks were eligible for inclusion. Consequently, women who had completely discontinued sexual activity, including those with potentially more severe sexual dysfunction, were not represented. This may have introduced selection bias and resulted in an underestimation of the true burden of sexual dysfunction. In addition, the sensitive nature of the study topic may have influenced participant recruitment and response accuracy, as some individuals may have been reluctant to disclose sexual concerns. Moreover, sexual function, fatigue, depressive symptoms, and quality of life were assessed using validated self-report questionnaires, which may be subject to recall and social desirability bias. Although multivariable analyses were performed, residual confounding due to unmeasured variables cannot be excluded. Finally, hormonal status, menopausal characteristics, and partner-related factors were not evaluated and may have influenced sexual function independently of COPD.

Despite these limitations, the study also has notable strengths. To our knowledge, this is one of the few studies specifically evaluating sexual dysfunction in women with COPD using validated instruments. Data were collected through structured face-to-face interviews under the supervision of multidisciplinary clinicians, enhancing the reliability of the findings. In addition, rigorous participant selection, including the restriction to nonsmokers and former smokers, minimized the potential confounding effects of tobacco exposure on sexual function. The comprehensive assessment, including clinical, psychological, and quality-of-life measures, allowed for a multidimensional evaluation of disease burden. Furthermore, the use of multivariable analysis strengthens the validity of the observed associations.

## Conclusions

This study found that sexual dysfunction in women with COPD was associated with fatigue, depressive symptoms, comorbidities, and greater disease burden (GOLD stage E), independent of age and spirometric severity. These findings suggest that sexual dysfunction may represent an additional aspect of COPD-related disease burden beyond airflow limitation alone. Assessment of sexual function may be considered as part of a more comprehensive evaluation of women with COPD. However, given the cross-sectional design of this study, no causal inferences can be drawn. Future prospective longitudinal studies are needed to confirm these associations, clarify causal relationships, and determine whether interventions targeting fatigue and depression improve sexual health and overall disease burden in this population.

### Clinical implications

Given the multifactorial nature of female sexual dysfunction in COPD, routine clinical assessment may benefit from incorporating brief screening for fatigue, depressive symptoms, and sexual concerns. A multidisciplinary approach involving pulmonologists, gynecologists, mental health professionals, and rehabilitation teams may provide a more comprehensive framework for addressing these complex and interrelated factors. Integrating sexual health counseling into pulmonary rehabilitation programs may represent a feasible strategy to improve overall quality of life.

## Supporting information

S1 Table Regression model diagnostics and collinearity diagnostics for the final multivariable linear regression model. (DOCX)
