# Supplementary material for: Association between sexual dysfunction and symptom burden in women with Chronic Obstructive Pulmonary Disease
Source: PLoS One. 2026 Sep 15;21(9):e0357921. doi: 10.1371/journal.pone.0357921 (PMC13577334; doi:10.1371/journal.pone.0357921)
Supplement: Supplementary file 1 [file pone.0357921.s001.docx]

Supplementary Table S1

| **Parameter** | **Value** |
| --- | --- |
| Number of observations | 100 |
| Regression method | Backward stepwise linear regression |
| Dependent variable | FSFI score |
| R² | 0.575 |
| Adjusted R² | 0.543 |
| F statistic | F(7,92) = 17.78 |
| Model p-value | <0.001 |
| Durbin-Watson statistic | 1,83 |
| VIF range | 1,08-1,47 |
| Tolerance range 0,68-0,93 |  |
|  |  |
| Residual diagnostics : Model assumptions were assessed by residual plots; no evidence of serious violations was observed. |  |

Collinearity Diagnostics

| **Variable** | **VIF** | **Tolerance** |
| --- | --- | --- |
| BMI | **1.08** | **0.93** |
| GOLD stage | **1.47** | **0.68** |
| FACIT-FS | **1.39** | **0.72** |
| Beck Depression Inventory | **1.21** | **0.83** |
| Dyspnea during intercourse | **1.38** | **0.72** |
| Comorbidity | **1.20** | **0.83** |
| Triple Therapy | **1.08** | **0.93** |
